# Do septins have a role in cancer?

**DOI:** 10.1038/sj.bjc.6602753

**Published:** 2005-09-05

**Authors:** S E H Russell, P A Hall

**Affiliations:** 1 Centre for Cancer Research & Cell Biology, Queen's University Belfast, University Floor, Tower Block, Belfast City Hospital, Belfast BT9 7AB, UK

**Keywords:** septin, cytoskeleton, scaffold, neoplasia, GTPase

## Abstract

Septins are an evolutionarily conserved family of genes that encode a P loop-based GTP-binding domain flanked by a polybasic domain and (usually) a coiled-coil region. They have roles in cytokinesis, vesicle trafficking, polarity determination, and can form membrane diffusion barriers, as well as in microtubule and actin dynamics. Septins can form hetero-oligomeric complexes and possibly function as dynamic protein scaffolds. Recently, it has been shown that there are at least 13 human septin genes that exhibit extensive alternate splicing. There are complex patterns of human septin gene expression and recently it has been found that alterations in septin expression are seen in human diseases including neoplasia. This review summarises the essential properties of septins and outlines the accumulating evidence for their involvement in human neoplasia. Septins may belong to the class of cancer critical genes where alteration in expression profile (including alterations in the spectrum of transcripts expressed) may underpin their role in neoplasia as opposed to specific mutational events.

In 1973, a group of cytokinesis mutants were identified in budding yeast by Hartwell. The protein products of these genes (*ScCdc3, ScCdc10, ScCdc11* and *ScCdc12*) localise to filamentous structures in the bud neck in *Saccharomyces cerevisiae* and were named *septins* by the laboratory of John Pringle. Much progress has been made in defining the range of functions of yeast septins and in the past 5 years their role in disease states, including cancer, has become apparent (Hall and Russell, 2004). Septins appear to function in yeast as spatial landmarks, elements of the polarity determination apparatus and diffusion barriers (reviewed in Irazoqui and Lew, 2004; Finger, 2005). They all have a central P loop-based GTP-binding domain and there is evidence that septins form hetero- and homo-oligomeric structures and can form filaments, although their significance remains uncertain (Hall and Russell, 2004). However, post translational modifications such as phosphorylation (Dobbelaere *et al*, 2003) and protein–protein interactions (Casamayor and Snyder, 2003) are crucial to septin function. They have been suggested to form scaffold-like structures upon which other proteins bind, thus allowing proper spatial and temporal control of processes such as polarity determination and cytokinesis. The septins are evolutionarily conserved although, interestingly, septin-like sequences have not been identified in plants to date, and cannot be found in Dictyostelium. Furthermore, it is curious that the number of septin genes differs in phylogeny, with seven in yeast, five in Drosophila and only two in *Caenorhabditis elegans* (Hall and Russell, 2004). Considerable expansion of the number of septin genes is seen in vertebrates and 13 are now known in man (Hall *et al*, 2005). These septin genes are distributed widely in the human genome as might be expected from an evolutionarily ancient gene family, but there remains considerable sequence conservation as well as an extraordinary conservation of certain aspects of genomic architecture and gene control.

The nomenclature of mammalian septins has proven problematic but a uniform nomenclature has simplified a Babel-like array of terms (see Table 1 in Hall and Russell (2004) for the diverse septin aliases). The 13 known human septins have remarkable similarity, all having in their longest forms (see Figure 1) a central GTP-binding domain flanked by a polybasic region and a so-called septin unique domain (Versele and Thorner, 2005). The function of the GTP-binding domain remains controversial. Although there are similarities with the small rho-like GTPases, it is not clear that the GTP- or GDP-bound state of septin has true signalling properties. It may be that like other GTP-binding proteins, such as tubulin, the GTP (or GDP) status confers structural properties and may influence oligomerisation. The polybasic domain of SEPT4 has been shown to bind phosphoinositol phosphates, and a reciprocal relationship between GTP and PIP2 binding has been reported (Casamayor and Snyder, 2003). It may be that targeting of septins to membrane domains is relevant to some of their functions including the potential to act as a diffusion barrier in both yeast and mammalian cells (reviewed in Finger, 2005). Most, but not all, septins have C-terminal coiled-coil domains that fall into two groups by amino-acid sequence SEPT6, 8, 10, 11 and SEPT1, 2, 4, 5, 7 and 13 (Hall *et al*, 2005). In contrast, SEPT3, 9 and 12 have a shorter C terminus without a coiled coil. This recapitulates the budding yeast septins where *ScCdc10* has no coiled coil whereas the others do. Three of the human septins (SEPT4, 8 and 9) have long N-terminal extensions, which have regions rich in proline residues.

The complexity of this gene family is increased by the existence of alternate splicing in most human septins, which dramatically increases the number of potential isoforms expressed. In the most extreme case so far defined, *SEPT9*, six 5′ splice variants can combine a common core domain with three 3′ splice variants to give at least 18 transcripts (McIlhatton *et al*, 2001) encoding 15 polypeptides. The discrepancy between the number of transcripts and isoforms is explained by the existence of two different 5′ transcripts that encode the same polypeptide. Many of the known isoforms encode truncated versions of a particular septin, which may act as (regulatory) dominant negative forms whose levels might modulate complex formation. In addition, the extraordinary observation of multiple splice variants encoding the same polypeptide has also been reported for *SEPT8* and *SEPT6*. The genomic, transcriptional and isoform complexity, coupled with the sheer number of humans septins, has hindered progress in our understanding of this family of genes but some progress has recently been made.

Comprehensive expression profiling of all members of the human septin family indicates that some septins are expressed in all tissues (eg *SEPT9*), while others have restricted profiles, with, for example, *SEPT3* only being found in the brain (Hall *et al*, 2005). Alterations in septin expression are seen in cancer and in other disease states. However, despite the size of the data sets studied, the complexity of splicing indicates that this global analysis can only be viewed as a first approximation to the definition of septin expression in man. In particular, the current data are inadequate for the delineation of the potential array of septin hetero-oligomers that might exist. Proteomic and biochemical analyses of specific septins suggest that some specific complexes can form (Versele and Thorner, 2005) and that, for example, SEPT2, 6 and 7 can form a stoichiometric association. In addition, specific coregulation can occur and experimental knock down of one component of this complex by siRNA leads to loss of expression of the other proteins in this complex. Furthermore, in mice lacking *SEPT5*, there is compensatory alteration in other septins (reviewed in Hall and Russell, 2004). Such data indicate that understanding the nature of septin complexes and their regulation are a central issue in the field.

A further crucial issue is the definition of the functional properties of human septins and an understanding of their biochemical attributes. While originally identified as a consequence of cytokinesis defects, it is clear that even in yeast, septins seem to have multiple functions (Hall and Russell, 2004). It would not be surprising if the increased number and complex distribution of mammalian septins is associated with an increased range of cellular functions. Data from multiple sources indicate that human septins can interact with other septins, as well as with components of the cytoskeleton such as actin and tubulin. In addition, interactions with S100A4, BORG3 and components of the exocytosis pathway have been reported (reviewed in Hall and Russell, 2004) and one truncated SEPT4 isoform has been associated with the induction of apoptosis via an interaction with XIAP (see below). Finally, recent data suggest a link with the small rho GTPases since the N terminus of SEPT9 binds a rhoGEF (Nagata and Inagaki, 2005).

## SEPTINS AND CANCER

### Septins: the MLL connection

The first clues to the role of septins in neoplasia came from the observation that balanced translocations involving septin loci and the MLL locus on chromosome 11 were seen in leukaemia giving rise to chimeric fusion proteins where the N terminus of MLL was fused, in frame, to almost the entire open reading frame of SEPT9 (Osaka *et al*, 1999). Subsequently, it has been found that three other septins (SEPT5, SEPT6 and SEPT11) can form very similar fusion proteins with MLL again with the N-terminal moiety of MLL fused to almost the entire open reading frame of the partner septin (Taki *et al*, 1999; Ono *et al*, 2002; Kojima *et al*, 2004). MLL is a remarkably promiscuous gene, forming in-frame chimeras with more than 50 other genes. Current data suggest that these fusion partners fall into two distinct groups: those with a potent transactivation domain and those that possess potential oligomerisation motifs. The septins do not possess an activation domain but are believed to oligomerise, possibly via their coiled-coil domains. However, SEPT9 does not contain a C-terminal coiled coil and one must thus posit a role for an alternative domain in forming oligomers. Some recent data support the idea that oligomerisation by the septin moiety of MLL fusions is important (Ono *et al*, 2005) and also points to a possible role for the GTP-binding domain in the formation of dimers.

### Linking *SEPT9* and cancer

SEPT9 was linked to neoplasia by two other observations. Sorensen *et al* (2000) identified the murine *SEPT9* locus as a common integration site for the SL-3 retrovirus in T-cell lymphomas. Thus, insertional mutagenesis at this locus suggests that *SEPT9* can contribute to neoplasia. Independently, the human *SEPT9* locus at 17q25.3 was identified as a common site for allelic imbalance in sporadic ovarian (Russell *et al*, 2000) and breast cancer (Kalikin *et al*, 2000). While mutations have not been observed in the known open reading frames of *SEPT9*, there is now abundant evidence pointing to altered expression of *SEPT9* in ovarian (Burrows *et al*, 2003) and breast (Montagna *et al*, 2003) tumours. Indeed *SEPT9* overexpression has been observed in diverse tumour types (Scott *et al*, 2005a). Of note is the observation that neoplasia is associated not just with altered expression of SEPT9 but also by alterations in the expression of specific *SEPT9* transcripts with the *SEPT9_v4* transcript being predominant in normal tissues but being replaced by SEPT9_v4^*^ in tumours (Burrows *et al*, 2003; Scott *et al*, 2005b). These transcripts encode the same polypeptide but differ in their 5′ UTR sequences. The SEPT9_v4^*^ transcript appears to be translated more efficiently than the SEPT9_v4 transcript (Russell and McDade, unpublished), and thus this change in transcript profile has a profound effect on the level of this SEPT9_v4 protein isoform.

### Other septins and cancer

Several lines of evidence have suggested that SEPT4 may be involved in neoplasia. Tanaka *et al* (2002) identified two alternate splice variants of *SEPT4* (and named it *Bradeion*) by screening an expression library. While expression of these transcripts is generally restricted to the brain, in tumours ectopic expression is observed. Furthermore, ribozyme-mediated downregulation of these transcripts could inhibit growth and tumorigenesis of colorectal cancer *in vivo* and *in vitro* and might be a useful diagnostic target (Tanaka *et al*, 2002). Independently, Larisch *et al* (2000) observed that what is now known as a *SEPT4* transcript (and was previously named ARTS) could promote TGF beta-mediated apoptosis. It has been reported that the SEPT4 isoform encoded by this transcript binds to and can modulate the function of XIAP and thus promote apoptosis (Gottfried *et al*, 2004). Subsequent studies have suggested that the expression of this transcript (which is distinct from those reported by Tanaka *et al*) might function as a tumour suppressor since expression is lost in most cases of childhood ALL (Elhasid *et al*, 2004). As if the terminological morass of SEPT4 (which has been named H5, bradeion, Pnutl2, ARTS, MAART, hCDCrel-2 and Septin-M) were not enough, the situation has become more complex with the report of SEPT4 knockout mice, which have not yet been reported to be tumour prone (Ihara *et al*, 2005). The apparent absence of a tumour phenotype may reflect the nature of the gene-targeting events (in effect deleting the entire locus) rather than excluding a role for SEPT4 in neoplasia. Indeed, it again underscores the need for transcript-specific analysis of septins, as is the case for SEPT9.

Other septins have been linked to neoplasia and the first human septin to be systematically studied, SEPT2 (previously known as Nedd5), was shown to be required for cytokinesis and to bind actin and associate with focal adhesions. Recent data suggest that SEPT2 can have a role in chromosome congression and segregation and that altered expression of SEPT2 might promote abnormalities of these crucial processes, leading to disordered chromosomal dynamics, and underlie the development of aneuploidy (Spiliotis *et al*, 2005). Whether these data are relevant to human tumours is as yet uncertain but certainly deserves further study. Our current catalogue of septin expression changes seen in neoplasia (and other disease states) remains far from complete and the complex splicing events seen in the septins makes progress difficult. Nevertheless, the available data suggest that at least some septins can be implicated in human (and murine) neoplasia. The crucial question is how?

### How do septins contribute to neoplasia?

The role of septins in cytokinesis would lead to the notion that these proteins are involved in neoplasia by perturbing cell division in some way. While this idea deserves attention and is supported by the recent observations of Spiliotis *et al* (2005), other possible explanations for the role of septins in neoplasia might be considered. The observation that septins can be involved in membrane dynamics is of interest given the increasingly recognised role of enhanced membrane dynamics in cancer (Polo *et al*, 2004). Another tantalising observation is the suggestion that one isoform of SEPT4 (previously called ARTS) can promote apoptosis (discussed above). Loss of function of this isoform might then reduce apoptosis and promote increase in cell number. Another observation of relevance to a potential role in neoplasia is the association of septins with both the actin and tubulin cytoskeleton (Surka *et al*, 2002; Nagata *et al*, 2003). Recently, Chacko *et al* (2005) have shown that the increased expression of the SEPT9_v4 protein has potent effects on the phenotype of epithelial cells. This isoform induces marked morphological changes in cultured cells with the generation of dramatic actin reorganisation and the formation of actin-based projections. In addition, SEPT9_v4 expression promotes cell motility in both two- and three-dimensional assays, and expression of GV mutants of SEPT9_v4 (analogous to gain of function mutants in GTP binding such as G12V in *ras*) promotes motility and perturbs the directionality of movement. These latter data are complemented by Golgi reorientation assays that suggest that SEPT9_v4 can alter cell polarity. This is perhaps not surprising given the role of septins in determining polarity in yeast (Irazoqui and Lew, 2004) and Finger *et al* (2003) have shown that a nematode septin can profoundly influence directional movement of developing neurons. How SEPT9_v4 induces these phenotypes remains uncertain but it is of note that the normal association of other SEPT9 isoforms with filamentous structures is perturbed by SEPT9_v4 and the GV mutant thereof (Chacko *et al*, 2005). SEPT9_v4 is a truncated form of the predominant long versions of SEPT9 and the phenotypic effects of overexpression are consistent with a model of it acting as a dominant negative species. The association of septins with microtubules is also of interest and it may be that septins can modulate aspects of microtubule function. Some of the observations of Chacko *et al* (2005) point to this since (for example) polarity determination requires microtubule coordination as well as effects on the actin cytoskeleton. The possibility that SEPT9 (and possibly other septins) can alter microtubule dynamics has been proposed, and this may be of relevance to drug resistance to microtubule-acting drugs.

A final aspect of septin function that may be relevant to neoplasia is their formation of complex hetero-oligomeric structures (Versele and Thorner, 2005) and their association with the rho signalling pathway (Nagata and Inagaki, 2005). In yeast, it has been suggested that septins act as scaffolds for the recruitment and regulation of proteins involved in several processes. It may be that in man the complex array of septins and septin isoforms provides an array of cell type and context-specific spatial cues that similarly organise the spatial arrangement of other proteins, potentially in a highly regulatable manner. Perhaps the stoichiometry of particular septins and their isoforms can control such processes. Consequently, the alteration in the level of septins in cells may have profound effects. The observation that septin levels change in neoplasia and that the overexpression of one isoform (SEPT9_v4) can have profound effects fits this class of model.

## CONCLUSION

The past 25 years have seen an explosion in our understanding of the molecular events underpinning neoplasia and more than 200 genes that are mutated in human cancers have been described (Futreal *et al*, 2004). However, genes whose protein products appear, at least in some tumour types, to contribute to the neoplastic phenotype continue to be identified. While it is the case that mutations are crucial to the role of many oncogenes and tumour suppressor genes in neoplasia, it is becoming increasingly apparent that the neoplastic phenotype can be a consequence of alterations in gene expression, with haploinsufficiency being an increasingly common theme (Mao *et al*, 2004), coupled with environmental factors, often having a multifaceted spatial (Orimo *et al*, 2005) and temporal interplay (Cook *et al*, 2005). Furthermore, the surprising revelation of how relatively few genes we have in the human genome and the extent of alternate splicing that exists, highlights the possibility that the range of genes whose products will have a role in neoplasia will continue to grow as we develop a more detailed understanding of the molecular events that regulate cells. Moreover, in some situations, the distinction between oncogenes and tumour suppressor genes may become blurred with the diverse products of one gene having different and potentially opposing functions.

The septin family of genes exemplify some of these issues and indicate how complicated the next 25 years of cancer research might be. Without question, the septin family deserves more attention and recent data suggest intriguing connections with fields as diverse as polarity control, membrane dynamics and exocytosis, the cell cycle and motility and cell shape. The consideration of septin biology and its role in neoplasia will require new perspectives and approaches to the issues of protein levels and stoichiometry, the nature and distribution of protein complexes and will ultimately require a much higher resolution analysis and with new reagents and approaches. It is also conceivable that the manipulation of septin complexes in cells may provide new insights to therapeutic options. Finally, the study of septins in neoplasia and other diseases may illuminate the broader issues of septin function in mammalian cells.

## Figures and Tables

**Figure 1:**
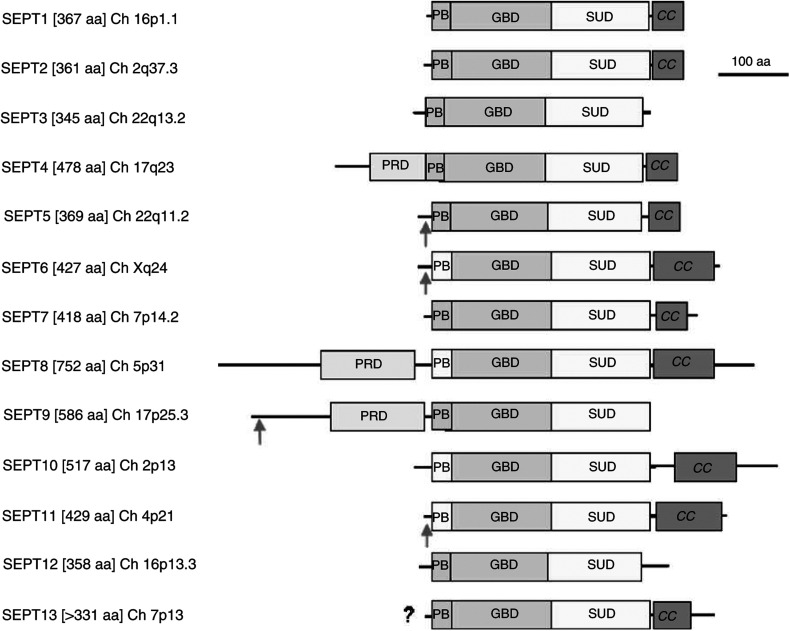
The human septins. The longest known versions of the 13 human septins described to date including their chromosomal location. Four human septins can form fusion proteins with the N-terminal moiety of MLL (arrowed). All have a polybasic domain (PB), although some are less basic (PB with lighter shade), a GTP-binding domain (GBD) and a septin unique domain (SUD). Some have a coiled-coil domain at the C terminus (CC). The longest forms of SEPT4, 8 and 9 have long N-terminal extensions with regions rich in prolines (PRD).
